# Construction of Immune-Related circRNA-miRNA-mRNA Network and Identification of circRNAs as Biomarkers in Coronary Atherosclerotic Heart Disease

**DOI:** 10.3390/cimb46110769

**Published:** 2024-11-13

**Authors:** Haiyan Qian, Yudan Chen, Jiali Chai, Keai Cheng, Xiya Zhao, Shuai Zhi, Yanqing Xie, Lina Zhang

**Affiliations:** 1 Health Science Center, Ningbo University, Ningbo 315211, China; qianhai202102@163.com (H.Q.); 13819996427@163.com (Y.C.); zhaoxiya2023@163.com (X.Z.); zhishuai@nbu.edu.cn (S.Z.); 2 Department of Cardiology, The First Affiliated Hospital of Ningbo University, Ningbo 315000, China; chaijiali1984@126.com (J.C.); 18358287580@163.com (K.C.)

**Keywords:** circular RNA, coronary atherosclerosis heart disease, biomarker, immune inflammation, regulatory network

## Abstract

Circular RNAs (circRNAs) play crucial roles in the immune and inflammatory responses of many diseases by acting as competing endogenous RNAs (ceRNAs). However, the role of circRNAs as ceRNAs in the immune and inflammatory processes of coronary atherosclerosis heart disease (CHD) remains unclear. This study aimed to identify and validate the potential immune-related circRNAs as biomarkers for CHD. Firstly, we constructed a ceRNA regulatory network including 14 circRNAs, 24 miRNAs, and 15 genes through bioinformatics analysis. Four hub genes were identified and five candidate immune-related circRNAs were screened. Subsequently, the expression levels of these candidate circRNAs were detected by qRT-PCR. Notably, hsa_circRNA_101069 and hsa_circRNA_406053 showed significant up-regulation in CHD patients (*p* < 0.001). The value of these circRNAs as biomarkers for CHD was evaluated by the area under the ROC curve (AUC), net reclassification improvement (NRI), and integrated discrimination improvement (IDI) indexes. Adding circRNAs to a traditional CHD model significantly enhanced classification performance, with an IDI of 0.058 and an NRI of 0.280 for hsa_circRNA_101069 and an IDI of 0.051 and an NRI of 0.480 for hsa_circRNA_406053. Furthermore, hsa_circRNA_101069 was up-regulated in ox-LDL-induced THP-1 macrophages, and silencing hsa_circRNA_101069 significantly inhibited the apoptosis rates and the inflammatory cytokines levels. This study constructed an immune-related circRNA-miRNA-mRNA network and identified two circRNAs as biomarkers for CHD, with hsa_circRNA_101069 potentially contributing to the pathological basis of CHD.

## 1. Introduction

Coronary atherosclerotic heart disease (CHD) is among the most prevalent cardiovascular diseases and poses a significant health and economic burden globally due to late diagnosis and serious complications [1]. Although the clinical treatment strategies for CHD continue to advance, the prognosis of patients remains suboptimal [2]. Currently, coronary angiography is the diagnostic “gold standard” for CHD, but its invasiveness and high cost limit its routine use in early clinical stages [3]. Thus, finding a convenient and accurate diagnostic method is critical for the early detection and timely treatment of CHD. The pathological basis of CHD lies in coronary atherosclerosis, a complex pathological process involving disrupted lipid metabolism and maladaptive immune responses that lead to chronic inflammation of the artery wall [4]. Growing evidence suggests that immunity and inflammation play important roles in the progression of atherosclerosis [5]. Oxidized low-density lipoprotein (ox-LDL) triggers the expression of leukocyte adhesion molecules and promotes the secretion of chemokines by endothelial cells, facilitating immune cell infiltration into the arterial intima [4]. Infiltrating monocytes differentiate into macrophages under the action of various cytokines, and then macrophages recognize and phagocytose lipids through scavenger receptors to form foam cells [6]. In addition, other immune cells such as mast cells and T lymphocytes also contribute to atherosclerosis development [7]. Based on the pathological characteristics of CHD, several inflammatory biomarkers (e.g., C-reactive protein, fibrinogen, serum amyloid A and interleukin-6) have been proposed for the diagnosis of CHD [8], but none have achieved effective clinical application. Therefore, it is urgent to identify novel immune/inflammatory markers for the rapid and accurate diagnosis of CHD.

Circular RNAs (circRNAs) are a new class of endogenous RNA molecules characterized by a covalently closed cyclic structure without 5ʹ caps or 3ʹ poly-A tails [9]. Because of their special cyclic structure, circRNAs are resistant to RNA exonuclease degradation and thus exhibit greater stability compared to linear RNAs [10]. CircRNAs are also characterized by high abundance, tissue/cell specificity, and evolutionary conservation [9]. Hence, circRNAs are expected to act as ideal biomarkers to diagnose diseases. Among the reported regulatory mechanism of circRNAs, the most common one is competing endogenous RNA (ceRNA). In short, circRNAs are rich in microRNA (miRNA) response elements and act as “miRNA sponges” to competitively bind to miRNAs, relieving the inhibitory effect of miRNAs on target genes and promoting the expression of target genes [11]. Accumulating evidence suggests that circRNAs serving as ceRNAs may fulfill immunoregulatory roles in some diseases. For example, circ_AFF2 acts as a “sponge” combined with miR-375, promotes the expression of TAB2, and then accelerates the inflammatory response of fibroblast-like synoviocytes in rheumatoid arthritis [12]. Hsa_circ_0087352 facilitates IL-6 transcription and the secretion of inflammatory cytokines by serving as a ceRNA for hsa-miR-149-5p in macrophages of abdominal aortic aneurysms [13]. However, there are few studies regarding circRNA serving as ceRNAs in the immune inflammation of coronary atherosclerosis.

**Figure 1:**
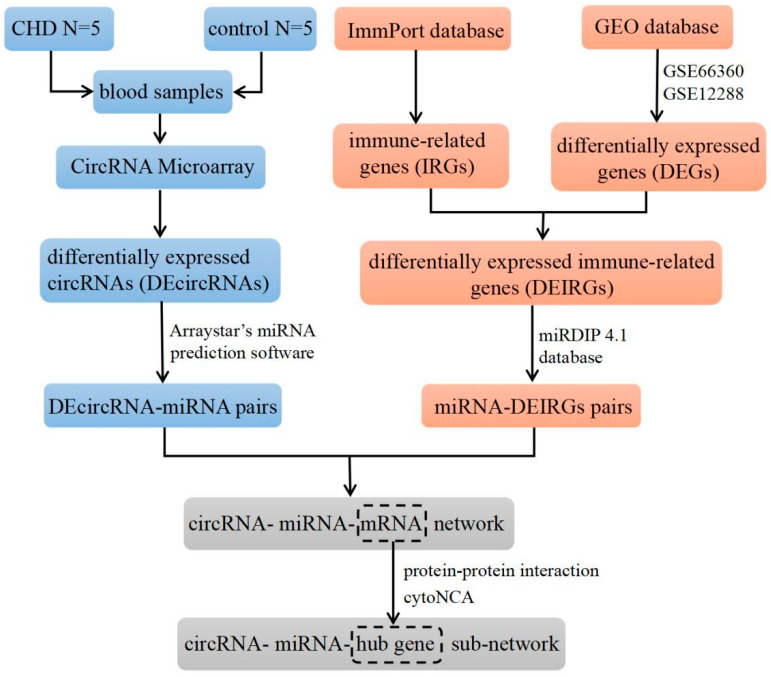
Flow chart for construction of immune-related circRNA-miRNA-mRNA network.

In this study, the Arraystar Human circRNA Array V2 analysis was used to obtain the circRNA microarray dataset of CHD, and the gene expression profile of CHD was derived from a public database. We first constructed an immune-related circRNA-miRNA-mRNA regulatory network by bioinformatics analysis, with the flow chart shown in Figure 1. Then, we carried out a case–control study to validate the expression levels of circRNAs and explored the association between immune-related circRNAs and CHD, aiming to identify novel biomarkers for early diagnosis. Additionally, the function of circRNA was verified in the human monocyte cell line THP-1.

## 2. Materials and Methods

### 2.1. Study Population

A hospital-based case–control study was conducted using a 1:1 matching method, with age (±3 years) and sex as matching variables. According to pre-experimental results, the exposure rate of the study factor (*P*_0_) was estimated to be 0.2, the odds ratio (OR) was estimated to be 2.6, *α* was 0.05, and *β* was 0.15, and the calculation showed that the sample size of a single group is 96. From January to December 2022, subjects were recruited from the First Affiliated Hospital of Ningbo University. Detailed demographic information, environmental factors, clinical parameters, pharmacological details, and diagnostic data for all subjects were collected using the hospital’s electronic case system and questionnaire.

Inclusion criteria were as follows: (a) the CHD group included patients diagnosed via coronary angiography showing ≥50% stenosis in at least one of the three main coronary arteries or main branches, and (b) the control group included subjects that were free of CHD after a series of assessments, including history inquiries and clinical examinations.

Exclusion criteria included congenital heart disease, valvular disease, myocarditis, severe heart failure, malignant arrhythmia, history of coronary revascularization procedures, pacemaker implantation, hepatic or renal dysfunction, autoimmune diseases, malignancies, serious brain organic diseases, and acute or chronic inflammatory diseases.

Following sex and age (±3 years) matching, 105 CHD patients and 105 non-CHD controls were enrolled. Of these, 5 CHD patients and 5 non-CHD controls were included in the circRNA microarray profiling phase, and 100 CHD patients and 100 non-CHD controls participated in the validation phase. Overnight fasting blood samples were drawn from each subject’s antecubital veins with ethylenediaminetetraacetic acid (EDTA) anticoagulated vacutainers. This study was conducted according to the Declaration of Helsinki (1975) and approved by the Ethics Committee of the First Affiliated Hospital of Ningbo University (KS20228006). All participants provided informed consent.

### 2.2. CircRNA Microarray Expression Profiling

Total RNAs from blood samples (5 CHD patients and 5 non-CHD controls) were extracted for circRNA microarray analysis. The concentration and purity of RNA were detected using a NanoDrop ND-1000 spectrophotometer (Nanodrop Technologies, Wilmington, DE, USA). The integrity of RNA was assessed by denaturing agarose gel electrophoresis. The total RNAs were digested, enriched, amplified, and transcribed into fluorescent cRNA based on the manufacturer’s standard protocol (Arraystar Inc., Rockville, MD, USA). The purified cRNAs were hybridized onto a microarray (Arraystar Human circRNA Array V2). Agilent Scanner G2505C was used to scan the hybridized arrays. Data from the acquired images were extracted by Agilent Feature Extraction software (version 11.0.1.1). Quantile normalization of raw data and differential analysis were performed using the “limma” package of R software. Differentially expressed circRNAs (DEcircRNAs) were selected through threshold with *t*-test *p*-value < 0.05 and |fold change| (FC) ≥ 1.5.

### 2.3. Identification of Differentially Expressed Immune-Related Genes

From the Gene Expression Omnibus (GEO) database [14] (https://www.ncbi.nlm.nih.gov/geo/, accessed on 1 October 2022), we retrieved and downloaded the CHD gene expression profile datasets (GSE66360 and GSE12288). GSE66360 [15] was based on the GPL570 [HG-U133_Plus_2] Affymetrix Human Genome U133 Plus 2.0 Array and contained samples from 49 CHD patients and 50 non-CHD controls. GSE12288 [16] was based on the GPL96 [HG-U133A] Affymetrix Human Genome U133A Array and included samples from 110 CHD patients and 112 non-CHD controls. Two datasets were combined after gene probe conversion and the batch effect was eliminated by the Combat function in the “sva” package of R. The “limma” package was applied to screen differentially expressed genes (DEGs) and the threshold was set as *p*-value < 0.05 and |FC| ≥ 1.2. A list of immune-related genes (IRGs) containing 1793 immune molecules was then downloaded from the ImmPort database [17] (https://www.immport.org/home). The overlapped part between DEGs and IRGs was obtained by a Venn diagram, that is, the differentially expressed immune-related genes (DEIRGs).

### 2.4. Functional and Pathway Enrichment Analysis

To assess the possible function of DEIRGs, gene ontology (GO) and Kyoto Encyclopedia of Genes and Genomes (KEGG) pathway enrichment analyses were performed using the DAVID Bioinformatics Resources [18] (https://david.ncifcrf.gov/). GO and KEGG terms with *p*-values below 0.05 were considered significant. The results were visualized by the “ggplot2” package in R.

### 2.5. Construction of the ceRNA Regulatory Network

Target miRNAs of the DEcircRNAs were predicted using Arraystar’s homemade miRNA target prediction software, establishing DEcircRNA-miRNA interaction pairs. And miRNA-DEIRGs interaction pairs were screened using the miRDIP 4.1 database [19] (http://ophid.utoronto.ca/mirDIP/) under a very high confidence filter (score class 1% = very high). Then, these DEcircRNA-miRNA pairs and miRNA-DEIRGs pairs were combined by overlapping the miRNAs to form an initial circRNA-miRNA-mRNA network. As circRNAs could competitively bind miRNA to promote the expression of target genes, only circRNAs and mRNAs with consistent expression direction were retained to construct the final ceRNA regulatory network. Cytoscape software (version 3.9.1) was used to visualize the ceRNA regulatory network.

### 2.6. Protein–Protein Interaction Network Analysis

A protein–protein interaction (PPI) network of the mRNAs in the ceRNA regulatory network was established by the Search Tool for the Retrieval of Interacting Genes (STRING) database [20] (https://cn.string-db.org/). The interaction score greater than 0.4 was set as the threshold. The results of PPI were visualized by Cytoscape software (version 3.9.1). In addition, in order to select the hub genes, the topological characteristics, including betweenness, closeness, and degree were analyzed by cytoNCA (version 2.1.6) plugin.

### 2.7. Cell Culture and Treatment

The human monocyte cell line THP-1 was purchased from HyCyte company (HyCyte™, Suzhou, China). THP-1 cells were cultured in RPMI 1640 medium (Gibco, Carlsbad, CA, USA) supplemented with 10% fetal bovine serum (FBS, Vivacell, Shanghai, China) and 1% penicillin/streptomycin (TransGen Biotech, Beijing, China) at 37 °C incubator with 5% CO_2_. THP-1 cells were seeded into six-well plates and treated with 100 ng/mL phorbol 12-myristate 13-acetate (PMA, Sigma, MO, USA) for 24 h to differentiation into macrophages. To establish a cell model of atherosclerosis, THP-1 macrophages were stimulated by 100 μg/mL ox-LDL (Yiyuan Biotechnologies, Guangzhou, China) for 48 h to form foam cells.

### 2.8. RNA Extraction and Quantitative Real-Time Reverse Transcription Polymerase Chain Reaction (qRT-PCR)

Total RNAs were isolated from the blood samples and THP-1 macrophages with TRIzol™ kit (Bioteke, Beijing, China) and converted to cDNA using GoScript™ RT Reagent Kit (Promega, Madison, WI, USA). The expression levels of the circRNAs were measured by qRT-PCR using the LightCycler 480II Real-Time PCR System (Roche, Rotkreuz, Switzerland). Specific primers (Table S1) were designed to amplify products. All samples were detected in triplicates. The human *GAPDH* was used as internal control. Relative expression levels were calculated by the 2^−ΔΔCT^ method.

### 2.9. Cell Transfection

Small interfering RNA (siRNA) targeting hsa_circRNA_101069 and negative control were synthesized via Genepharma (Shanghai, China). THP-1 macrophage was transiently transfected using Lipofectamine 3000 (Invitrogen, CA, USA), and the efficiency was assessed by qRT-PCR analysis. The sequences of siRNAs are shown in Table S2.

### 2.10. Enzyme-Linked Immunosorbent Assay (ELISA)

The supernatant of the cells was collected to detect the concentrations of tumor necrosis factor-α (TNF-α), interleukin-6 (IL-6), and interleukin-1β (IL-1β) using specific ELISA kits (Proteintech, Wuhan, China) following the manufacturer’s instructions. The optical density (OD) was measured at 450 nm by Multiskan GO microplate reader (Thermo Fisher, Waltham, MA, USA).

### 2.11. Flow Cytometry Assay

The cell apoptosis rate was detected using the Annexin V-FITC/PI apoptosis kit (MultiSciences, Hangzhou, China). Briefly, the cell suspension was stained with 10 µL PI and 5 µL Annexin V-FITC for 5 min in the dark and then detected by the CytoFlex S flow cytometer (Beckman Coulter, CA, USA).

### 2.12. Statistical Analysis

All statistical analyses were carried out using SPSS 21.0 (IBM, Chicago, IL, USA), GraphPad Prism 8.0 (GraphPad Software, San Diego, CA, USA) and R software (version 4.2.1). According to whether the data obeyed the normal distribution, continuous variables were presented as the mean ± standard deviation or median (first quartile, third quartile), and differences between two groups were examined by Student’s *t*-test or the Mann–Whitney *U* test. Analysis of variance (ANOVA) and multiple comparisons were used to analyze the differences among multiple groups of quantitative variables. Categorical variables were presented as frequency and percentage (%) and differences between two groups were examined by *χ^2^* test. Correlation analyses of clinical parameters and circRNAs were performed using Spearman’s rank correlation test. Logistic regression models were applied to assess whether candidate circRNAs were independent influencing factors for CHD. The diagnostic potentiality of circRNAs for CHD was assessed by receiver operating characteristic (ROC) curve analysis. The area under the ROC curve (AUC) between different models was compared by the DeLong test. Reclassification analysis was used to evaluate the improvement of the model added circRNAs by net reclassification improvement (NRI) and integrated discrimination improvement (IDI) indexes. All tests were performed as two-sided and *p*-values lower than 0.05 were considered statistically significant.

## 3. Results

### 3.1. Identification of DEcircRNAs and DEIRGs in CHD

**Figure 2:**
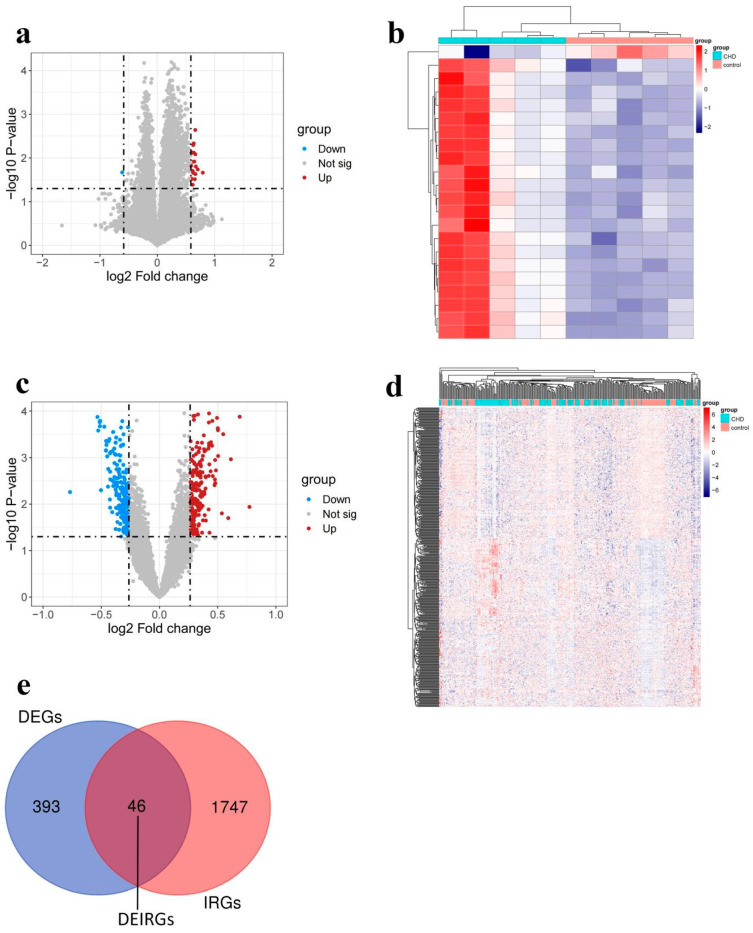
DEcircRNAs, DEGs, and DEIRGs in CHD. Volcano plot of (**a**) DEcircRNAs and (**c**) DEGs. Red and blue dots represent upregulation and downregulation, respectively. Heatmap of (**b**) DEcircRNAs and (**d**) DEGs. The color from red to navy represents a trend from high expression to low expression. (**e**) Venn diagram of DEIRGs. Blue circle represents DEGs, red circle represents IRGs, and their overlapped part represents DEIRGs.

To screen immune-related candidate circRNAs in CHD, we analyzed the circRNA microarray expression profile and gene expression profile datasets. A total of 22 DEcircRNAs (21 up-regulated and 1 down-regulated) were identified in the circRNA microarray expression profile (Figure 2a,b). A total of 439 DEGs (229 up-regulated and 210 down-regulated) were obtained from the GSE66360 and GSE12288 datasets (Figure 2c,d). The detailed results of DEcircRNAs and DEGs were listed in Tables S3 and S4. A total of 1793 IRGs were obtained from the ImmPort database, and then 46 DEIRGs were generated by the overlapped part between DEGs and IRGs (Figure 2e).

### 3.2. Functional and Pathway Enrichment Analysis

**Figure 3:**
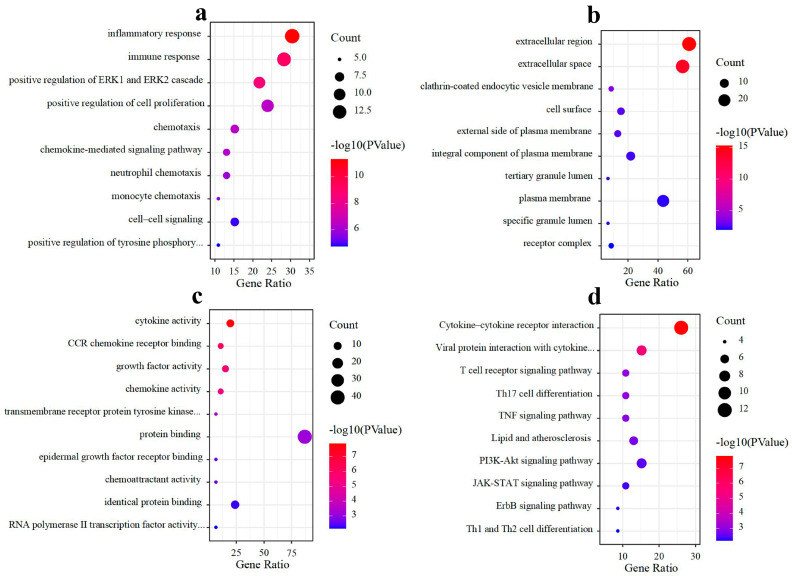
Top ten significantly enriched terms by GO and KEGG analysis. (**a**) Biological process, (**b**) cellular component, (**c**) molecular function, and (**d**) KEGG pathway.

To understand the potential function of DEIRGs, GO and KEGG pathway enrichment analyses were conducted. Based on the results of GO analysis, the top ten significantly enriched terms of biological process (BP), cellular component (CC), and molecular function (MF) are shown in Figure 3a–c, respectively. Among them, the BP included inflammatory response, immune response, positive regulation of ERK1 and ERK2 cascade, and so on; the CC was mainly extracellular region and extracellular space; the MF was related to cytokine activity, CCR chemokine receptor binding, growth factor activity, and so on. The results of KEGG pathway analysis mainly enriched in the PI3K-Akt signaling pathway and the JAK-STAT signaling pathway, and other pathways are shown in Figure 3d.

### 3.3. Construction of the ceRNA Regulatory Network

**Figure 4:**
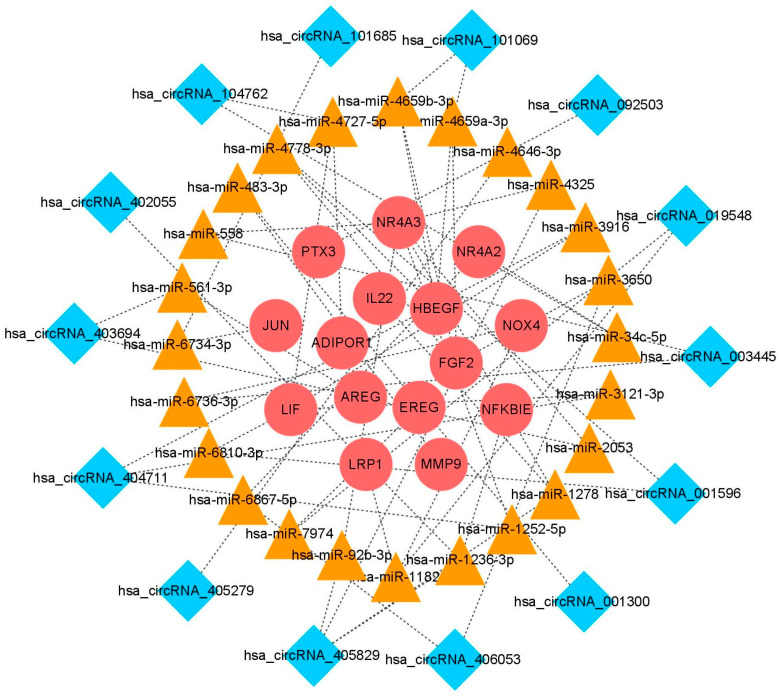
The ceRNA regulatory network. Blue diamonds represent circRNAs, orange triangles represent miRNAs, and red circles represent genes.

To explore the potential relationship between DEcircRNAs and DEIRGs, we established a regulatory network based on the ceRNA hypothesis. A total of 110 DEcircRNA-miRNA pairs and 1087 miRNA-DEIRGs pairs were established, respectively, using Arraystar’s homemade miRNA target prediction software and the miRDIP 4.1 database. Then, DEcircRNA and DEIRGs were screened by overlapping the miRNAs. Finally, a ceRNA regulatory network with 53 nodes and 56 edges was constructed, comprising 14 DEcircRNAs, 24 miRNAs, and 15 DEIRGs (Figure 4).

### 3.4. PPI Analysis and Sub-Network Construction

**Figure 5:**
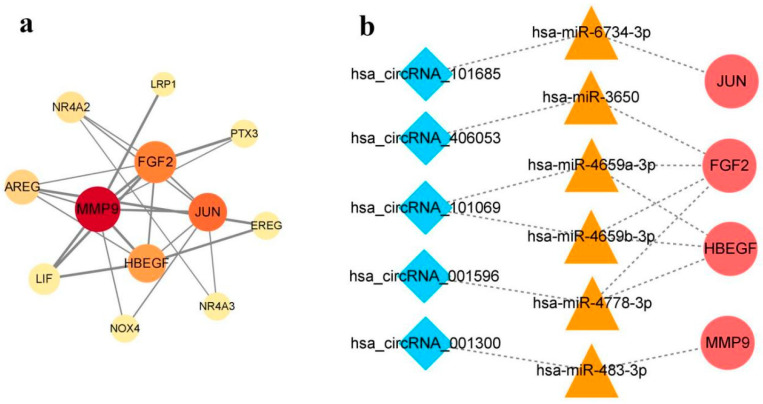
PPI network and circRNA-miRNA-hub gene sub-network. (**a**) PPI network consisting of DEIRGs in the ceRNA network. The color (from yellow to red) and size of node represent their betweenness and degree in network, respectively. The width of edge represents the combined score between DEIRGs. (**b**) circRNA-miRNA-hub gene sub-network extracted from the ceRNA network. Blue diamonds represent circRNAs, orange triangles represent miRNAs, and red circles represent genes.

A PPI network was established to illustrate the interactions among the 15 DEIRGs in the ceRNA network using the STRING database. This PPI network was visualized by Cytoscape software and contained 12 nodes and 23 edges after removing the unconnected nodes (Figure 5a). According to topological features (betweenness, closeness, and degree), four DEIRGs had values above the average levels calculated on the PPI network, identifying them as potential hub genes (Table 1).

Based on the aforesaid four hub genes, a circRNA-miRNA-hub gene sub-network was extracted from the ceRNA regulatory network, comprising five circRNAs, six miRNAs, and four genes nodes (Figure 5b). Since these nodes may be the most valuable in the ceRNA regulatory network, we hypothesized that these five circRNAs (hsa_circRNA_101069, hsa_circRNA_101685, hsa_circRNA_406053, hsa_circRNA_001300, and hsa_circRNA_001596) could be candidate biomarkers of CHD for further study.

### 3.5. Clinical Characteristics of the Subjects

The clinical characteristics of the subjects were detailed in Table 2. Statistically significant differences were observed between the two groups concerning smoking, drinking, hypertension, diabetes, platelet-to-lymphocyte ratio (PLR), neutrophil-to-lymphocyte ratio (NLR), monocyte-to-lymphocyte ratio (MLR), glycated hemoglobin (GHb), alkaline phosphatase (AKP), total cholesterol (TC), low-density lipoprotein (LDL), and homocysteine (HCY) (*p* < 0.05). No significant differences were observed in other variables (*p* > 0.05).

### 3.6. Validation of the Candidate circRNAs in Population Samples

**Figure 6:**
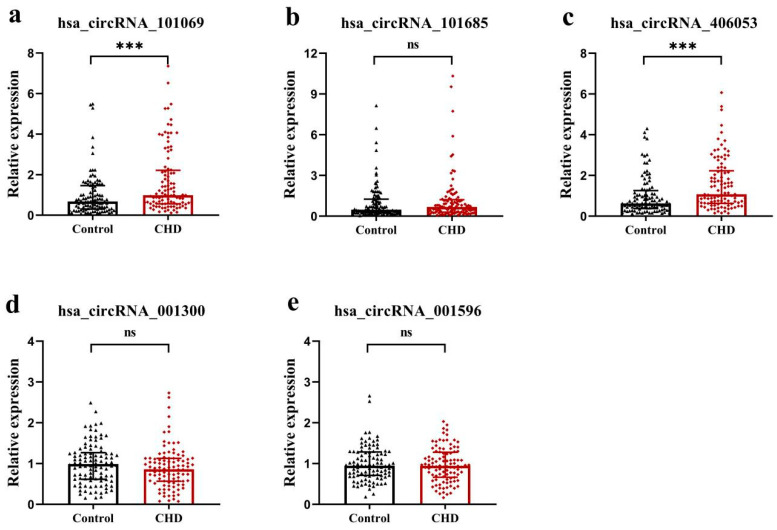
qRT-PCR analysis of expression levels of circRNAs. (**a**) hsa_circRNA_101069, (**b**) hsa_circRNA_101685, (**c**) hsa_circRNA_406053, (**d**) hsa_circRNA_001300, and (**e**) hsa_circRNA_001596. Data were presented as the median and interquartile range, using Mann–Whitney *U* test. ns, no significant. *** *p* < 0.001.

To validate the dysregulation of candidate circRNAs in CHD patients compared to controls, we performed a qRT-PCR to detect their relative expression levels. The expression levels of circRNAs hsa_circRNA_101069 and hsa_circRNA_406053 were significantly increased in CHD patients compared to controls (*p* < 0.001) (Figure 6a,c), consistent with the results of circRNA microarray analysis. No significant differences were observed in the expression levels of hsa_circRNA_101685, hsa_circRNA_001300, and hsa_circRNA_001596 between the two groups (*p* > 0.05) (Figure 6b,d,e).

### 3.7. Identification of hsa_circRNA_101069 and hsa_circRNA_406053 as Independent Influencing Factors of CHD

Univariate and multivariate logistic regression analyses were performed to identify whether hsa_circRNA_101069 and hsa_circRNA_406053 were independent risk factors for CHD. As shown in Table 3, the influence of hsa_circRNA_101069 and hsa_circRNA_406053 on CHD was statistically significant after adjusting for smoking, drinking, hypertension, and diabetes (*p* < 0.05).

### 3.8. Potential of hsa_circRNA_101069 and hsa_circRNA_406053 as Biomarkers for CHD

**Figure 7:**
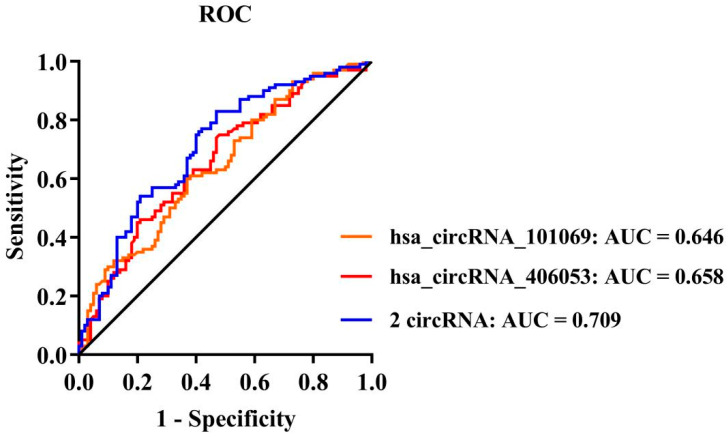
ROC curve of circRNAs.

The results of ROC analysis showed that hsa_circRNA_101069 and hsa_circRNA_406053 were statistically significant for distinguishing CHD from the control population (Figure 7). The AUC increased to 0.709 when two circRNAs were combined. The AUC, specificity, and sensitivity were detailed in Table S5.

Additionally, we evaluated the role of adding two circRNAs to a clinical model including the traditional risk factors of CHD: smoking, drinking, hypertension, and diabetes (Table 4). The clinical model was used as the reference model, with an AUC of 0.678 (95% *CI*: 0.604–0.752, *p* < 0.05). Incorporating hsa_circRNA_101069 into the model significantly increased the AUC compared to the clinical model alone (*p* < 0.05). The addition of hsa_circRNA_101069 showed a statistically significant reclassification of the population samples with IDI of 0.058 (95% *CI*: 0.026–0.091, *p* < 0.001) and NRI of 0.280 (95% *CI*: 0.020–0.540, *p* < 0.05). Similarly, adding hsa_circRNA_406053 to the clinical model resulted in a statistically significant reclassification (IDI = 0.051, 95% *CI*: 0.020–0.081, *p* < 0.05; NRI = 0.480, 95% *CI*: 0.224–0.736, *p* < 0.001), though the AUC was not significantly increased (*p* > 0.05). Adding hsa_circRNA_101069 and hsa_circRNA_406053 to the clinical model at the same time showed a statistically significant reclassification and significantly improved the discrimination of the clinical model (Table 4). By contrast, adding hs-CRP did not improve either the AUC or reclassification (Table 4).

### 3.9. Correlation of hsa_circRNA_101069 and hsa_circRNA_406053 with Inflammation Index

To further explore the clinical significance of hsa_circRNA_101069 and hsa_circRNA_406053, we evaluated the correlation between their expression levels and inflammation indexes. Spearman’s rank correlation analysis revealed a significant correlation between the hsa_circRNA_101069 expression level and NLR (*p* < 0.001), and between the hsa_circRNA_406053 expression level and PLR, NLR, and MLR (*p* < 0.001) (Table 5).

### 3.10. Effect of Silencing hsa_circRNA_101069 in THP-1 Macrophages

**Figure 8:**
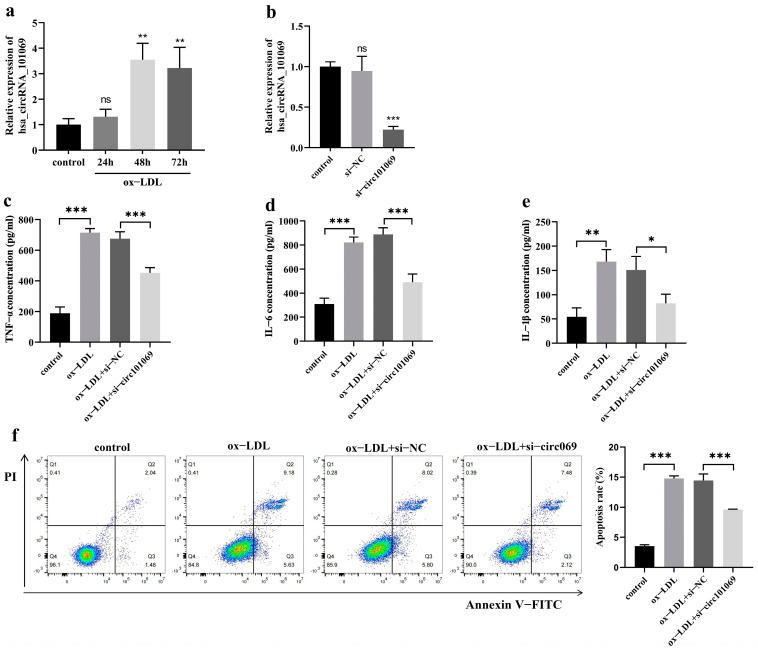
Effect of silencing hsa_circRNA_101069 in THP-1 macrophages. (**a**) The expression level of hsa_circRNA_101069 in THP-1 macrophages. (**b**) The expression level of hsa_circRNA_101069 in THP-1 macrophages after siRNA transfection. (**c**–**e**) The concentrations of TNF-α, IL-6, and IL-1β measured by ELISA. (**f**) The cell apoptosis rates measured by flow cytometry. Data were from three independent experiments and presented as the mean ± standard deviation, using ANOVA and Dunnett-*t* (**a**,**b**) or Tukey’s (**c**–**f**) test. ns, no significant. * *p* < 0.05, ** *p* < 0.01, *** *p* < 0.001.

Since atherosclerosis is the pathological basis of CHD, THP-1 macrophages were induced by ox-LDL to establish a cell model. The qRT-PCR analysis showed that hsa_circRNA_101069 was up-regulated in ox-LDL-induced THP-1 macrophages compared to the control (Figure 8a). Then, we explored the effects of silencing hsa_circRNA_101069 in THP-1 macrophages. The expression level of hsa_circRNA_101069 was significantly suppressed after siRNA transfection (Figure 8b). Ox-LDL promoted the cell inflammation to increase inflammatory cytokine, while silencing hsa_circRNA_101069 significantly inhibited the levels of TNF-α, IL-6, and IL-1β (Figure 8c–e). Furthermore, silencing hsa_circRNA_101069 partly attenuated the apoptosis induced by ox-LDL in THP-1 macrophages (Figure 8f).

## 4. Discussion

CHD is an atherosclerotic cardiovascular disease with high morbidity and mortality worldwide. Recent studies have highlighted the role of immunity and inflammation in the pathological basis of CHD [21]. Therefore, the identification of novel immune/inflammatory biomarkers in coronary atherosclerosis is of great significance for diagnosing and treating CHD. CircRNA is a novel endogenous RNA molecule with a covalently closed-loop structure [9]. With the development of high-throughput sequencing technology, more circRNAs have been discovered in organisms, and their important biological functions have been gradually revealed [22]. The function of circRNA as ceRNA is the most common one. For instance, circRNA_010567 accelerates myocardial fibrosis via promoting TGF-β1 production as a ceRNA for miR-141 [23]. In regard to the pathological basis of CHD, circ_0001785 can reduce endothelial cell injury and thus delay atherogenesis through the miR-513a-5p/TGFBR3 ceRNA network mechanism [24].

In this study, we first identified 22 DEcircRNAs and 439 DEGs from the circRNA microarray expression profile and gene expression profile datasets, respectively. Based on the pathological characteristic of chronic inflammation in coronary atherosclerosis, we aimed to identify immune/inflammatory markers in CHD. Thus, we further identified 46 DEIRGs from DEGs. Through functional enrichment analysis with these 46 DEIRGs, some pathways drew our attention, such as the PI3K-Akt signaling pathway and the JAK-STAT signaling pathway. The PI3K-Akt signaling pathway plays a key role in the survival, proliferation, and migration of macrophages and thus influences the development of atherosclerosis [25]. The knockdown of CXCR4 alleviates the degree of CHD by promoting macrophage autophagy through the PI3K/AKT/mTOR pathway to reduce atherosclerosis [26]. Additionally, the activation of the JAK/STAT signaling pathway generates vascular inflammation and regulates key atherosclerotic processes by inducing the expression of inflammatory genes [27]. Another noteworthy pathway identified was the pathway of lipid and atherosclerosis. The above results suggested that these 46 DEIRGs are likely involved in the pathological process of CHD through regulating immune inflammation response.

Based on the ceRNA hypothesis, circRNAs could competitively bind miRNAs to regulate the expression of target genes [11]. We predicted the interactions between the RNAs using bioinformatics databases and then established a circRNA-miRNA-mRNA network composed of 14 DEcircRNAs, 24 miRNAs, and 15 DEIRGs. We established a PPI network of fifteen DEIRGs and then screened out four hub genes, including MMP9, FGF2, JUN, and HBEGF. MMP9, a member of the peptidase M10A family, plays an important role in the proteolysis of the extracellular matrix and leukocyte migration. MMP-9 is involved in endothelial dysfunction in several ways, such as increasing the migration and invasion into the arterial wall of inflammatory cells, participating in the ox-LDL effect and endothelial apoptosis, and can be a biomarker of endothelial dysfunction in atherosclerotic plaque instability [28]. FGF2 is a ligand for FGFR1, FGFR2, FGFR3, and FGFR4. Liu et al. [29] concluded that FGF2 and its receptors have dual roles for the cardiovascular system: their expression is beneficial to vascular homeostasis, vascular protection, and endothelial survival in the normal vessel wall, while in atherosclerotic processes, they participate in the inflammatory response, intimal thickening, and intra-plaque angiogenesis. JUN is one of the transcription factor AP-1. Shan et al. [30] demonstrated that Angiotensin II specifically regulates the expression of macrophage migration inhibitory factors in human umbilical vein endothelial cells via the transcription factor AP-1, which results in the destabilization of atherosclerotic plaque. HBEGF, secreted from human macrophages, is a potent mitogen for vascular smooth muscle cells (SMCs) and fibroblasts [31]. As early as the 1990s, scholars have found that HBEGF is closely associated with coronary atherogenesis through playing roles in the interaction between SMCs and macrophages, the migration of SMCs to the intima, and the proliferation of SMCs [32].

After mapping the four hub genes to the circRNA-miRNA-mRNA network, we extracted a circRNA-miRNA-hub gene sub-network. The five circRNAs in this sub-network are likely to be immune-related circRNAs involved in the pathological process of CHD. A subsequent analysis showed the differential expression of hsa_circRNA_101069 and hsa_circRNA_406053 between CHD patients and controls. Furthermore, the high expression of hsa_circRNA_101069 and hsa_circRNA_406053 was an independent risk factor for CHD after adjusting for potential confounders. CircRNA is easier to detect and recognize in blood samples due to its better stability, longer half-life, and tissue/cell specificity [33]. Hence, circulating circRNAs are suitable as novel biomarkers for the prediction and diagnosis of certain diseases including cardiovascular disease, such as heart failure [34], dilated cardiomyopathy [35], and CHD [36]. Although previous studies have shown the potential of circRNAs as biomarkers for CHD, less attention has been devoted to immune-related circRNAs involved in the pathological process of CHD. We thus further explored the diagnostic value of hsa_circRNA_101069 and hsa_circRNA_406053 as biomarkers for CHD. Both hsa_circRNA_101069 and hsa_circRNA_406053 were statistically significant in discriminating CHD patients from the control population, but the discriminative power was low. When these two circRNAs were combined, the discriminative power improved to a moderate level. The occurrence of CHD is affected by both genetic and environmental factors [37], and the combination of epigenetic makers and traditional cardiovascular risk factors can improve their application value in the risk assessment of CHD patients. NRI and IDI have been proposed as alternatives to the increase in the AUC for evaluating the improvement in the performance of risk assessment algorithms brought about by adding new markers [38]. Wang et al. [39] found that the addition of miR-423-3p together with the traditional risk factors markedly improved the model performance measures, including the AUC and NRI. In this study, we first constructed a clinical model to predict CHD based on the traditional risk factors of CHD (smoking, drinking, hypertension, and diabetes). The AUC, NRI, and IDI were not improved by the addition of hs-CRP. Adding hsa_circRNA_101069 and hsa_circRNA_406053 to the clinical model alone or in combination significantly improved the reclassification performance for CHD. We thereby provided evidence that hsa_circRNA_101069 and hsa_circRNA_406053 could be novel biomarkers in CHD prediction. In recent years, several inflammatory markers from the whole blood count, including PLR, NLR, and MLR have attracted extensive attention of scholars [40]. PLR, NLR, and MLR have been shown to predict the severity of atherosclerosis in CHD. NLR and MLR have important prognostic significance on long-term survival after off-pump coronary artery bypass grafting [41]. In our study, we found that the hsa_circRNA_101069 expression level was correlated with NLR, and the hsa_circRNA_406053 expression level was correlated with PLR, NLR, and MLR, further supporting their potential as biomarkers for CHD.

Coronary atherosclerosis is the pathological basis of CHD. Monocytes and macrophages play central roles in the atherosclerotic process [42]. Monocytes in the arterial intima mature into macrophages, which can further produce multiple inflammatory cytokines [43]. We preliminarily investigated the functional significance of hsa_circRNA_101069 in THP-1 macrophages by RNA interference, which revealed that silencing hsa_circRNA_101069 inhibited the apoptosis rates and TNF-α, IL-6 and IL-1β concentrations in ox-LDL-induced THP-1 macrophages. Hence, it could be speculated that hsa_circRNA_101069 might contribute to the pathogenesis of atherosclerosis.

It should be acknowledged that there are some limitations to our study. First, this is a single-center retrospective case–control study, so the role of circRNAs as biomarkers of CHD needs to be fully validated in a multicenter large prospective study. Second, our functional exploration of hsa_circRNA_101069 in THP-1 macrophages was preliminary. The mechanism regulating the pathological basis of CHD through the circRNA-miRNA-mRNA axis needs to be further verified in vivo and in vitro. Future research will aim to explore the specific pathways of miRNAs and downstream mRNAs targeted by circRNA_101069 in THP-1 macrophages, which could provide evidence for circRNAs as potential therapeutic targets in the pathological process of CHD.

## 5. Conclusions

In summary, our study constructed an immune-related circRNA-miRNA-mRNA regulatory network and identified that hsa_circRNA_101069 and hsa_circRNA_406053 were closely associated with CHD, suggesting their potential as biomarkers for CHD prediction. Additionally, we found the function of hsa_circRNA_101069 in THP-1 macrophages, providing preliminary evidence for its involvement in atherosclerosis pathogenesis.

## Figures and Tables

**Table 1:** Four hub genes identified by cytoNCA.

Gene	Betweenness	Closeness	Degree	Gene Descriptions
*MMP9*	38.00	0.79	16	Matrix metalloproteinase-9
*FGF2*	20.67	0.73	14	Fibroblast growth factor 2
*JUN*	23.67	0.69	12	Transcription factor AP-1
*HBEGF*	15.67	0.69	12	Proheparin-binding EGF-like growth factor

**Table 2:** Clinical characteristics of the subjects.

Variables	Control (N = 100)	CHD (N = 100)	*χ*^2^/*t*/*Z*	*p*
Basic information				
Gender (male)	60 (60.0)	60 (60.0)	-	-
Age (years)	61.16 ± 10.77	62.19 ± 11.04	-	-
Smoking (*n*, %)	36 (36.0)	50 (50.0)	3.998	**0.046**
Drinking (*n*, %)	18 (18.0)	30 (30.0)	3.947	**0.047**
BMI (Kg/m^2^)	24.70 ± 3.32	25.15 ± 3.84	−0.849	0.397
Hypertension (*n*, %)	55 (55.0)	79 (79.0)	13.026	**<0.001**
Diabetes (*n*, %)	16 (16.0)	29 (29.0)	4.846	**0.028**
Clinical parameters				
PLR	120.21 (85.61, 147.83)	134.36 (106.36, 171.76)	−2.460	**0.014**
NLR	2.19 (1.54, 2.87)	2.70 (2.04, 3.66)	−3.377	**0.001**
MLR	0.21 (0.15, 0.27)	0.22 (0.18, 0.31)	−2.164	**0.030**
hs-CRP (mg/L)	1.15 (0.40, 2.43)	1.50 (0.50, 3.20)	−0.396	0.692
BNP (ng/L)	13.40 (5.00, 42.23)	32.40 (9.60, 76.10)	−1.344	0.179
cTnI (ng/mL)	0.03 (0.00, 0.03)	0.03 (0.00, 0.03)	−1.773	0.076
GHb (%)	5.75 (5.50, 6.00)	6.00 (5.70, 6.95)	−2.596	**0.009**
AKP (U/L)	56.50 (47.50, 67.25)	68.00 (54.00, 78.00)	−3.690	**<0.001**
GT (U/L)	21.50 (16.75, 34.50)	31.00 (21.00, 66.00)	−1.866	0.062
TG (mmol/L)	1.08 (0.70, 1.46)	1.31 (0.96, 1.59)	−0.435	0.663
TC (mmol/L)	4.55 ± 1.09	4.18 ± 1.18	2.196	**0.029**
HDL (mmol/L)	1.27 ± 0.53	1.17 ± 0.36	1.520	0.130
LDL (mmol/L)	2.48 ± 0.75	2.19 ± 0.79	2.529	**0.012**
HCY (mmol/L)	8.65 (6.83, 11.00)	11.90 (7.95, 17.00)	−3.309	**0.001**

Abbreviations: BMI, body mass index; PLR, platelet-to-lymphocyte ratio; NLR, neutrophil-to-lymphocyte ratio; MLR, monocyte-to-lymphocyte ratio; hs-CRP, hypersensitive C-reactive protein; BNP, brain natriuretic peptide; cTnI, cardiac troponin I; GHb, glycated hemoglobin; AKP, alkaline phosphatase; GT, glutamyl transpeptidase; TG, triglyceride; TC, total cholesterol; HDL, high-density lipoprotein; LDL, low-density lipoprotein; HCY, homocysteine.

**Table 3:** Logistic regression analysis of circRNAs for CHD.

circRNA	Univariate		Multivariate	
	OR (95% *CI*)	*p*	^a^ OR (95% *CI*)	^a^ *p*
hsa_circRNA_101069	1.514 (1.167–1.964)	**0.002**	1.514 (1.140–2.009)	**0.004**
hsa_circRNA_406053	1.633 (1.203–2.216)	**0.002**	1.500 (1.099–2.046)	**0.011**

Note: **^a^** is adjusted for smoking, drinking, hypertension, and diabetes. OR, odds ratio; *CI*, confidence interval.

**Table 4:** Potential of circRNAs as biomarkers for CHD.

Model	Discrimination		Reclassification			
	AUC (95% *CI*)	^a^ *p*	IDI (95% *CI*)	*p*	NRI (95% *CI*)	*p*
Clinical Model (CM)	0.678 (0.604–0.752)	-	Reference model	-	Reference model	-
CM + hs-CRP	0.688 (0.615–0.762)	0.289	0.013 (−0.003–0.029)	0.107	0.020 (−0.215–0.255)	0.868
CM + hsa_circRNA_101069	0.730 (0.660–0.799)	**0.043**	0.058 (0.026–0.091)	**<0.001**	0.280 (0.020–0.540)	**0.035**
CM + hsa_circRNA_406053	0.723 (0.653–0.793)	0.061	0.051 (0.020–0.081)	**0.001**	0.480 (0.224–0.736)	**<0.001**
CM + 2 circRNA	0.748 (0.680–0.815)	**0.017**	0.087 (0.048–0.127)	**<0.001**	0.640 (0.388–0.893)	**<0.001**

Note: **^a^** is vs. clinical model. Clinical model (CM): smoking, drinking, hypertension, and diabetes. hs-CRP, hypersensitive C-reactive protein; IDI, integrated discrimination improvement index; NRI, net reclassification improvement index.

**Table 5:** Correlation between circRNAs and inflammation indexes.

Inflammation Index	hsa_circRNA_101069		hsa_circRNA_406053	
	*S_r_*	*p*	*S_r_*	*p*
PLR	0.017	0.809	0.260	**<0.001**
NLR	0.345	**<0.001**	0.337	**<0.001**
MLR	0.010	0.888	0.280	**<0.001**

Abbreviations: PLR, platelet-to-lymphocyte ratio; NLR, neutrophil-to-lymphocyte ratio; MLR, monocyte-to-lymphocyte ratio.

## Data Availability

The data that support the findings of this study are available from the corresponding author upon reasonable request.

## Supplementary Materials

The following supporting information can be downloaded at https://www.mdpi.com/article/10.3390/cimb46110769/s1, Table S1: Specific primers for qRT-PCR analysis; Table S2: The sequences of siRNAs; Table S3: Differentially expressed circRNAs; Table S4: Differentially expressed genes; Table S5: The results of ROC curve analysis.
