# Supplementary material for: Construction of Immune-Related circRNA-miRNA-mRNA Network and Identification of circRNAs as Biomarkers in Coronary Atherosclerotic Heart Disease
Source: Curr Issues Mol Biol. 2024 Nov 13;46(11):12914–30. doi: 10.3390/cimb46110769 (PMC11592537; doi:10.3390/cimb46110769)
Supplement: Supplementary file 1 [file cimb-46-00769-s001.zip › cimb-3278912-supplementary.pdf]

Table S1. Specific primers for qRT-PCR analysis.

Primer name	Primer sequence (5' - 3')
hsa_circRNA_101069-F	CGTCCTGCTACAGACAGCTT
hsa_circRNA_101069-R	TCAGCCAGAAAGTCTGTTAGG
hsa_circRNA_101685-F	AAGGAGGCTGAAGGGTACATC
hsa_circRNA_101685-R	GCATCGGCATGGTACTCCA
hsa_circRNA_406053-F	GAGGTGAAGAGGGAGGATAT
hsa_circRNA_406053-R	GCTGTCAAGTTACCACTTCC
hsa_circRNA_001300-F	TAGAGGCGGGTGGTTACATT
hsa_circRNA_001300-R	CTATCCAGCCTCCTTCTGTA
hsa_circRNA_001596-F	AAAGGCCAAGAAGGCTGCA
hsa_circRNA_001596-R	GGAGTCTTCTTCACTGCCT
GAPDH-F	TGTTGCCATCAATGACCCCTT
GAPDH-R	CTCCACGACGTACTCAGCG

F: forward primer. R: reverse primer.

Table S2. The sequences of siRNAs.

siRNA name	sense (5' - 3')	antisense (5' - 3')
si-circ101069	UUUGGAGACACAAAAAAGATT	UCUUUUUUGUGUCUCCAAATT
si-NC	UUCUCCGAACGUGUCACGUTT	ACGUGACACGUUCGGAGAATT

Table S3. Differentially expressed circRNAs.

circRNA	circBase ID	*P*	Fold change	Regulation
hsa_circRNA_406825		0.021693396	1.7327938	up
hsa_circRNA_019548	hsa_circ_0019548	0.007461286	1.5155041	up
hsa_circRNA_404711		0.041029104	1.5331146	up
hsa_circRNA_101069	hsa_circ_0026497	0.021901555	1.5412602	up
hsa_circRNA_001300	hsa_circ_0001393	0.029339139	1.5063437	up
hsa_circRNA_402055		0.002284834	1.5817108	up
hsa_circRNA_405829		0.008206386	1.5839169	up
hsa_circRNA_092503	hsa_circ_0001129	0.019439818	1.5158848	up
hsa_circRNA_406053		0.012264863	1.53512	up
hsa_circRNA_405279		0.005056733	1.5431647	up
hsa_circRNA_101685	hsa_circ_0037516	0.02333576	1.5856202	up
hsa_circRNA_406955		0.004641261	1.5525558	up
hsa_circRNA_104120	hsa_circ_0076742	0.007557081	1.553746	up
hsa_circRNA_001596	hsa_circ_0001596	0.030637901	1.5720566	up
hsa_circRNA_003445	hsa_circ_0003445	0.007940305	1.501456	up
hsa_circRNA_001166	hsa_circ_0001556	0.012060897	1.5638174	up
hsa_circRNA_007738	hsa_circ_0007738	0.015116076	1.5048494	up
hsa_circRNA_104762	hsa_circ_0003141	0.01568577	1.5924159	up
hsa_circRNA_405788		0.030864084	1.5060871	up
hsa_circRNA_403694		0.018273225	1.6293001	up

hsa_circRNA_000446	hsa_circ_0001664	0.020208488	1.536932	up
hsa_circRNA_050054	hsa_circ_0050054	0.021402675	1.52744	down

Table S4. Differentially expressed genes.

Gene	*P*	Fold change	Regulation
S100P	0.000000124	1.730665136	up
NR4A2	0.000000907	1.667349829	up
CDA	0.00000111	1.386442323	up
CXCL2	0.00000112	1.760705421	up
MGP	0.00000129	1.652429257	up
ADIPOR1	0.00000343	1.273925537	up
RGS1	0.00000569	1.780195857	up
SPI1	0.00000584	1.342609034	up
PLAU	0.00000589	1.410563253	up
MS4A4A	0.00000655	1.391989823	up
MMP9	0.000012	1.378622827	up
MAFF	0.0000127	1.509082183	up
TULP2	0.0000137	1.445531246	up
EFEMP1	0.0000271	1.599498411	up
GADD45A	0.000036	1.343675787	up
TMEM158	0.0000526	1.511942685	up
NCAPH	0.0000645	1.33127342	up
MAFB	0.0000645	1.50724885	up
BTNL8	0.0000974	1.436588724	up
CD68	0.000112516	1.342872469	up
HIST1H2AG	0.000118492	1.257788615	up
RP11-457I16.2	0.000125959	1.233600909	up
SERPINB8	0.000127816	1.223102659	up
PTX3	0.000132706	1.611782328	up
JUN	0.00014048	1.408392347	up
HK3	0.000154688	1.227496389	up
KIAA1598	0.000163297	1.369682468	up
LIN7A	0.000176222	1.341479154	up
BCAT1	0.000234707	1.41531279	up
EMP1	0.000258671	1.41646481	up
AREG	0.000315022	1.461630467	up
ASGR2	0.000333425	1.301921645	up
RBM38	0.000446627	1.203798414	up
GAB2	0.000468343	1.358909849	up
NFKBIE	0.000478455	1.253433367	up
MEIS1	0.000480797	1.287504081	up

CDKN1A	0.000495284	1.343306823	up
DENND1A	0.000549711	1.34289267	up
DOCK4	0.000552124	1.367073025	up
CRABP2	0.000589107	1.245362477	up
SERPING1	0.000624236	1.269304169	up
CCL14	0.000634585	1.338453592	up
NRGN	0.000655599	1.30348096	up
KCNJ15	0.000657593	1.215755312	up
MARC1	0.000693623	1.316088443	up
RP11-473I1.9	0.000729658	1.307541381	up
FCAR	0.000736971	1.26151588	up
PRG3	0.000757144	1.262745282	up
CNGB3	0.000841715	1.242505917	up
SIGLEC5	0.000912419	1.294900607	up
LRRC32	0.000914394	1.224565264	up
HIST1H2AE	0.000919343	1.417623449	up
CCL3	0.001080591	1.529665544	up
MIR631	0.001095448	1.207384547	up
LILRA2	0.001101325	1.204512039	up
LOC101928916	0.001104767	1.289013176	up
NR4A3	0.0011217	1.39274586	up
KDM6B	0.001168846	1.215539199	up
BFSP2	0.001230444	1.272140421	up
CREM	0.001274637	1.2299884	up
MEIS3P1	0.00134257	1.269911884	up
SNIP1	0.001361566	1.230937724	up
ACSL1	0.001377314	1.26167669	up
CH25H	0.001392944	1.345676381	up
HOXA10	0.001400213	1.277440684	up
HIVEP1	0.001467001	1.245457059	up
GINS4	0.00152828	1.280000452	up
DDIT3	0.001731138	1.247957475	up
DBF4B	0.001875223	1.224355442	up
IFI27	0.001875549	1.399186137	up
MBOAT2	0.001887479	1.23537082	up
FFAR2	0.001904046	1.221660749	up
SMAD1	0.001967248	1.356039398	up
DAAM2	0.001990291	1.244506018	up
GPR15	0.001999468	1.27251396	up
PADI2	0.002062003	1.291912484	up
AZGP1P1	0.002118119	1.246990647	up
STYK1	0.002132013	1.201425151	up
SIRPB1	0.002147506	1.27767298	up
LRP1	0.002185082	1.220538283	up

PDZD8	0.002373855	1.25345483	up
VWF	0.002376567	1.258250782	up
PIR	0.002463321	1.3401445	up
PI3	0.002470899	1.276688653	up
LOC100129518	0.002477172	1.21823476	up
SPARCL1	0.002480808	1.373228036	up
BMX	0.002534262	1.314495016	up
IRAK3	0.00264259	1.316950193	up
PTRF	0.0026705	1.277016464	up
ELAVL2	0.002937742	1.390857877	up
IDI2-AS1	0.003331821	1.202365661	up
SH3TC2	0.003371222	1.284540136	up
UTY	0.003375577	1.210399395	up
NT5DC2	0.003485557	1.24417928	up
PHACTR1	0.003527205	1.281950981	up
CD33	0.003533434	1.275679429	up
MYL9	0.003537167	1.393839085	up
SOX4	0.00370942	1.208524672	up
CYP4F3	0.003714185	1.22915415	up
CCRL2	0.003742052	1.270813104	up
TMEM51	0.003764122	1.245871387	up
ADIRF	0.003812001	1.260853437	up
PIGH	0.003863108	1.248127492	up
EREG	0.003907016	1.387832995	up
HBEGF	0.004020792	1.263352848	up
SYNDIG1	0.004297725	1.224399771	up
NECAB2	0.004469301	1.218617333	up
DSC2	0.004604064	1.315513839	up
AOC3	0.004657702	1.243173152	up
FCGR1A	0.004854009	1.217486359	up
ARG1	0.00495201	1.266225283	up
SLC7A5	0.005006909	1.231132042	up
GABRA2	0.005115091	1.245566877	up
TNFAIP6	0.005418289	1.305185785	up
LHFP	0.005466178	1.284381388	up
HIST1H3B	0.005489198	1.282981688	up
IL22	0.005987521	1.210096652	up
LIMCH1	0.006061881	1.306979469	up
OSM	0.00607053	1.281531703	up
CHI3L1	0.006083862	1.284054639	up
PFKFB4	0.006159088	1.208877758	up
ALDH1A1	0.006219331	1.289484848	up
LY96	0.006383327	1.210854611	up
S100A12	0.006628823	1.273728047	up

CFD	0.006690983	1.212566579	up
PIGA	0.006763328	1.288809545	up
CBX4	0.006971634	1.28120185	up
KIR2DL5A	0.007256949	1.209547365	up
FCN1	0.007396467	1.20867432	up
CAMSAP2	0.007588471	1.235419518	up
CDH8	0.007773857	1.241055434	up
CHRNA5	0.007837143	1.210236102	up
XYLT1	0.007870513	1.223797145	up
ORC1	0.007973746	1.206122011	up
IL1R2	0.008274685	1.24346908	up
SLC1A3	0.008298749	1.219761912	up
GRM2	0.008340105	1.205355864	up
FEZF2	0.0083448	1.255246636	up
ADRA1B	0.008735685	1.220519496	up
SRGAP2	0.009203569	1.200969745	up
MIR22	0.009534776	1.265771899	up
EPAS1	0.009572651	1.262144604	up
ORM1	0.009661263	1.274795051	up
BCL2A1	0.009730908	1.222268988	up
TRMT44	0.009845327	1.233945929	up
HIST3H3	0.009902131	1.215319541	up
FGF2	0.009914381	1.254384446	up
CYR61	0.0099764	1.245969359	up
ACSM3	0.010374075	1.207082861	up
NUPL1	0.01061809	1.253607525	up
NLRP3	0.010858288	1.292388607	up
RIMBP2	0.011126422	1.254621955	up
IL2	0.011306546	1.262900439	up
RPS4Y1	0.011459521	1.708569942	up
EGR3	0.011797804	1.281432922	up
FCGR3A	0.012097569	1.214141391	up
SLPI	0.012388372	1.201142149	up
SPOCK3	0.012388506	1.276001006	up
BST1	0.012401593	1.201019286	up
CLU	0.012463756	1.212933381	up
BC069782	0.012708454	1.261083301	up
PLSCR4	0.012997421	1.33974212	up
TFPI	0.013582802	1.265200517	up
PLA2G4A	0.013947641	1.236368032	up
FCGR1B	0.014019632	1.212127889	up
CSN1S1	0.014038668	1.204488749	up
RP1-68D18.4	0.014814552	1.210950219	up
OLR1	0.014911571	1.200669287	up

ASGR1	0.014913932	1.239753964	up
HIST1H2BN	0.015587685	1.220223469	up
FGA	0.015757373	1.202083587	up
EIF1AY	0.015791842	1.449696485	up
KIAA1462	0.016137812	1.208303534	up
AIF1	0.016432137	1.206808078	up
GFM1	0.016621513	1.224780162	up
KRT85	0.016660008	1.235031396	up
PLK2	0.016865462	1.211383066	up
NOX4	0.017053235	1.263565594	up
ADAM9	0.017118992	1.23495778	up
RPE65	0.01726148	1.308878641	up
BUB1	0.017472285	1.241591812	up
SEC61A2	0.018892034	1.20609318	up
USP6NL	0.019195352	1.214203632	up
C16orf95	0.019387488	1.221328209	up
HTRA1	0.019423291	1.229195076	up
DDX3Y	0.019933876	1.505930302	up
SLC24A3	0.020834836	1.224197189	up
PCDH17	0.021451911	1.213769971	up
BCHE	0.022227007	1.200714124	up
TBCAP2	0.022279235	1.295311796	up
GREB1L	0.022444162	1.211987811	up
ZBTB10	0.02252957	1.317845431	up
MEIS2	0.022627004	1.226873039	up
CDH5	0.02287201	1.205139796	up
LOC100507472	0.024325594	1.213311235	up
MYH4	0.025009081	1.214998721	up
SIK1	0.025096276	1.216494256	up
ANXA3	0.025438481	1.205646426	up
MUC7	0.025641906	1.202780499	up
EGR2	0.026716391	1.207521457	up
EGR1	0.026981937	1.264981603	up
CDKL3	0.027580377	1.231706329	up
CCDC68	0.028557011	1.204033894	up
LOC100127886	0.028957041	1.26160144	up
OR12D3	0.02936387	1.200423348	up
NDUFAF5	0.029372555	1.205693315	up
LIF	0.029391698	1.208728445	up
KIF24	0.029613836	1.218393218	up
SPATA6	0.030309292	1.22791934	up
CCL20	0.031412695	1.212918974	up
UGT2B28	0.033513582	1.228209679	up
ZNF80	0.03473216	1.247645554	up

CDADC1	0.035149309	1.2220201	up
GC	0.036261996	1.201204307	up
TMEM100	0.037141533	1.232525925	up
MLF1	0.038350072	1.207198635	up
KIF18A	0.039293354	1.2510877	up
SERPINB2	0.040444058	1.22437772	up
SIX3	0.040952527	1.234979974	up
TMEM144	0.04108962	1.288336309	up
SULF1	0.041159742	1.251050506	up
TMEM176B	0.04118547	1.34673455	up
SLC19A2	0.042871593	1.209656807	up
ERC2-IT1	0.043077898	1.233409398	up
TFEC	0.043921971	1.213431626	up
SKIL	0.044318856	1.248818995	up
LOC101928274	0.045938007	1.218301142	up
FAM198B	0.046026531	1.257671327	up
GAS2	0.047512986	1.216688856	up
RIC8B	0.00000266	1.376185859	down
ANKRD46	0.0000161	1.357122458	down
RP11-119F7.5	0.0000485	1.476801608	down
GZMA	0.0000528	1.28590149	down
CAND2	0.0000564	1.408657941	down
DYRK4	0.0000613	1.33533688	down
TBCCD1	0.0000726	1.349322917	down
METTL2A	0.000134111	1.446865394	down
GIN1	0.000162727	1.424165076	down
ZCCHC10	0.000164951	1.247916722	down
MINPP1	0.000173214	1.427914177	down
ACADSB	0.000211814	1.426202907	down
KIZ	0.000217377	1.389239799	down
FLT3LG	0.000224005	1.205861176	down
ARL2	0.000226601	1.257402707	down
CRTAM	0.000262537	1.440256722	down
CASP8AP2	0.000283032	1.317573718	down
MAGEF1	0.000316551	1.238898707	down
LINC00260	0.000370985	1.294652306	down
CCDC7	0.000401229	1.346762076	down
SH3YL1	0.000414166	1.265202391	down
KIAA1024	0.00043942	1.374398734	down
PTPN13	0.000444437	1.354138371	down
C17orf80	0.0004772	1.298978403	down
ELP4	0.000479115	1.297962726	down
LOC100506403	0.000526941	1.241472913	down
PRR5L	0.000532338	1.371305463	down

SMYD5	0.000546839	1.253661916	down
KLHL36	0.000564782	1.258733873	down
ARMCX5	0.000634189	1.309120374	down
SAC3D1	0.000691174	1.375452908	down
LRRC37A2	0.000705437	1.375021808	down
CDR2	0.000719824	1.323435454	down
GALNT12	0.000756091	1.336998774	down
SACS	0.000804431	1.342811473	down
PTGDR	0.00083168	1.304328254	down
PTCH1	0.00086756	1.29841199	down
MAPKBP1	0.000872766	1.272150283	down
GPR157	0.000896871	1.262701326	down
ZNF83	0.000938827	1.3654108	down
ATF1	0.001157751	1.266817744	down
NIF3L1	0.001303116	1.307366503	down
MROH7-TTC4	0.001426264	1.240555177	down
PKIA	0.001429009	1.287675093	down
CETN3	0.00174652	1.356890129	down
ASUN	0.001762349	1.270401684	down
SAP30L	0.001776693	1.29162987	down
CASP10	0.00180038	1.216728057	down
CD3G	0.002000446	1.341383896	down
MSH2	0.002052414	1.274112555	down
SPECC1L	0.002057507	1.241078412	down
NRIP1	0.002094738	1.220316243	down
OSGEPL1	0.002324402	1.268468917	down
ATF6	0.002586164	1.261489385	down
EXOC5	0.002605002	1.307931461	down
NPAS2	0.002643741	1.235392263	down
ANKRD36	0.00272664	1.296977166	down
LOC202181	0.002731276	1.30117795	down
TTC27	0.002760096	1.281512761	down
C12orf29	0.002936182	1.339771864	down
SMARCAL1	0.002949283	1.229475073	down
ZNF557	0.003080631	1.324804464	down
TTC1	0.003326385	1.257405196	down
KCNQ1OT1	0.003336983	1.313032316	down
MFHAS1	0.003385715	1.297195717	down
COLQ	0.003446304	1.282099826	down
GAL3ST4	0.003461384	1.232441602	down
ADAM22	0.00347412	1.250374635	down
GTF2H2B	0.003502383	1.202502437	down
TMEM168	0.003618681	1.222117049	down
ZNF268	0.003879318	1.294747534	down

FRS2	0.004071136	1.317129741	down
ZNF177	0.004103232	1.287630644	down
BPNT1	0.00420219	1.359006955	down
KLHL3	0.004232918	1.207419012	down
WNT16	0.004276944	1.248201767	down
HOPX	0.004313407	1.217358185	down
PPP1R3A	0.00440635	1.26101063	down
NOS3	0.00444083	1.21394859	down
LOC157562	0.004577748	1.22572497	down
SMAGP	0.00462841	1.260651962	down
RUVBL1	0.004636616	1.228563256	down
PLA2G12A	0.004671252	1.208005999	down
ENOSF1	0.004717999	1.257709771	down
DCK	0.004732626	1.232062306	down
TARP	0.004740897	1.250292627	down
SLC44A1	0.004868812	1.298438613	down
AF070581	0.00500506	1.41663198	down
INSIG2	0.005036566	1.231489572	down
XIST	0.005491843	1.703360795	down
METTL21B	0.005530862	1.24942404	down
STXBP1	0.005621843	1.218096776	down
LIN7C	0.005704453	1.291989213	down
TTBK2	0.005743116	1.220363467	down
SUPT3H	0.006037157	1.230869855	down
MYBL1	0.006114052	1.238528169	down
MEX3C	0.006235025	1.231954063	down
DFFB	0.00630004	1.27157743	down
TBC1D31	0.006371901	1.27882905	down
KRBOX4	0.006408016	1.293759952	down
PDCD5	0.006425789	1.20318945	down
NUDT7	0.006773985	1.230098385	down
ANKEF1	0.00679168	1.26959292	down
SCRIB	0.007265943	1.209012545	down
HN1L	0.007464574	1.202139749	down
PIGK	0.007595397	1.2725806	down
CEL	0.007778289	1.203229283	down
MPHOSPH9	0.007956614	1.343677686	down
SLC4A7	0.007971988	1.227774564	down
MRPS28	0.008075046	1.242620079	down
CD40LG	0.008260334	1.255088216	down
SYCE1L	0.008338961	1.253822083	down
NEK1	0.008451104	1.215965732	down
MRPS33	0.008844952	1.243957537	down
ZNF137P	0.009186388	1.214076651	down

ZFP64	0.009231216	1.232062421	down
MGA	0.009274242	1.217933829	down
CCDC106	0.009360195	1.202168766	down
ABCB7	0.009361336	1.223304064	down
HSD17B2	0.009700869	1.223201548	down
ZNF248	0.010059574	1.203160402	down
EFNA4	0.010112868	1.218417211	down
CCL16	0.01026742	1.238199082	down
IGHG1	0.010418689	1.248170382	down
RNF144A	0.010598205	1.25306631	down
OR2A20P	0.010668937	1.272390814	down
ENDOG	0.01139983	1.211886555	down
MFSD6	0.011887739	1.341875213	down
SERPINI1	0.01197452	1.294966844	down
FSBP	0.012057047	1.27701921	down
TKTL1	0.012261473	1.27839716	down
CNPY4	0.01235639	1.234426551	down
PCYOX1	0.012448268	1.26380117	down
C11orf63	0.012456442	1.232818309	down
SETD4	0.012571099	1.201562843	down
RP11-15P13.1	0.013397854	1.262290431	down
LZTFL1	0.013493161	1.233925718	down
FBXL15	0.014205002	1.205097942	down
CLIC1P1	0.014738019	1.241358313	down
ZNF780B	0.01486825	1.324960462	down
SPAG16	0.014910108	1.265508906	down
MOCS2	0.014937055	1.229831882	down
DOCK9	0.015078371	1.231798954	down
KIR3DX1	0.015165624	1.211634016	down
TEF	0.015717407	1.228193461	down
IKZF3	0.015864016	1.21632418	down
LMAN2L	0.016259202	1.208187216	down
CDC7	0.016271737	1.24096589	down
GMEB1	0.016302927	1.208526728	down
NXPE3	0.016403387	1.285304225	down
TRMT13	0.016543446	1.247993279	down
WNT2B	0.016679479	1.211764291	down
TRNAU1AP	0.017153901	1.218815548	down
SPON1	0.017434142	1.214422191	down
PFAS	0.017450349	1.231976205	down
ORC3	0.018073807	1.239340784	down
BCOR	0.018476621	1.249532509	down
LNPEP	0.019035138	1.256868687	down
FASTKD1	0.019261313	1.202239465	down

TEFM	0.019380116	1.218641935	down
MTUS2	0.019804227	1.208512218	down
ANKH	0.019909952	1.247394264	down
NBEA	0.020010283	1.200584	down
RPL23AP22	0.020596033	1.243169296	down
PHTF2	0.021439937	1.210470755	down
AMIGO2	0.021542801	1.202578497	down
PPP4R4	0.022805239	1.233097917	down
ZNF443	0.023098094	1.207082911	down
NR3C2	0.023864496	1.232838206	down
AGPAT4	0.024047839	1.232806259	down
THNSL1	0.024315056	1.284678335	down
ZKSCAN4	0.024612137	1.210830037	down
MRPL35	0.024963684	1.209868684	down
ZNF550	0.025289796	1.204430853	down
PIBF1	0.026638423	1.262425531	down
AV8S2	0.026800194	1.214861987	down
ZFP37	0.027035567	1.213122892	down
ZSCAN26	0.027289027	1.203868158	down
CEP97	0.028091972	1.215854957	down
PNPLA3	0.028818202	1.205945475	down
CYP2E1	0.029310836	1.205382924	down
NRP2	0.030340162	1.208707703	down
GSTCD	0.030989395	1.203201126	down
LINC00094	0.03135533	1.222675473	down
ENPP4	0.031662374	1.234759424	down
HOMER1	0.032112287	1.23089401	down
DPY19L2P2	0.032135908	1.205418316	down
CEP72	0.032330956	1.204138437	down
SP4	0.033692559	1.202487678	down
B3GALT2	0.034321192	1.29810011	down
FANCF	0.034426535	1.240753181	down
TRAF3IP1	0.034567376	1.221578696	down
TUBGCP5	0.035592926	1.208098549	down
KIAA0754	0.035608223	1.271665548	down
HERC2	0.036921176	1.212338947	down
MYOT	0.037049925	1.236586004	down
EXOC2	0.038410798	1.205526443	down
RBL1	0.038592955	1.239849285	down
NUDT6	0.039366111	1.201262831	down
ZNF623	0.039405036	1.203971515	down
KLRAP1	0.039481891	1.238455197	down
ANAPC10	0.03976621	1.23768553	down
FZD7	0.040606871	1.233981519	down

MRPL42	0.040855442	1.209077749	down
IL24	0.041130901	1.20123544	down
MSANTD2	0.042998942	1.207853023	down
THAP1	0.04327132	1.205371759	down
PRDM13	0.044089808	1.21524914	down
ERCC4	0.048396838	1.200116553	down
FGFR3	0.04972743	1.256721537	down

Table S5. The results of ROC curve analysis.

circRNA	AUC（95% *CI*）	*P*	Sensitivity	Specificity
hsa_circRNA_101069	0.646 (0.570 - 0.722)	**<0.001**	0.61	0.62
hsa_circRNA_406053	0.658 (0.583 - 0.733)	**<0.001**	0.75	0.52
2 circRNA	0.709 (0.637 - 0.781)	**<0.001**	0.83	0.53
